# Comparing Effectiveness of Top-Down and Bottom-Up Strategies in Containing Influenza

**DOI:** 10.1371/journal.pone.0025149

**Published:** 2011-09-22

**Authors:** Achla Marathe, Bryan Lewis, Christopher Barrett, Jiangzhuo Chen, Madhav Marathe, Stephen Eubank, Yifei Ma

**Affiliations:** 1 Network Dynamics and Simulation Science Lab, Virginia Bioinformatics Institute at Virginia Tech, Blacksburg, Virginia, United States of America; 2 Department of Agricultural and Applied Economics, Virginia Tech, Blacksburg, Virginia, United States of America; 3 Department of Computer Science, Virginia Tech, Blacksburg, Virginia, United States of America; 4 Department of Physics, Virginia Tech, Blacksburg, Virginia, United States of America; Umeå University, Sweden

## Abstract

This research compares the performance of bottom-up, self-motivated behavioral interventions with top-down interventions targeted at controlling an “Influenza-like-illness”. Both types of interventions use a variant of the *ring strategy*. In the first case, when the fraction of a person's direct contacts who are diagnosed exceeds a threshold, that person decides to seek prophylaxis, e.g. vaccine or antivirals; in the second case, we consider two intervention protocols, denoted *Block* and *School*: when a fraction of people who are diagnosed in a Census *Block* (resp., *School*) exceeds the threshold, prophylax the entire *Block* (resp., *School*). Results show that the bottom-up strategy outperforms the top-down strategies under our parameter settings. Even in situations where the *Block* strategy reduces the overall attack rate well, it incurs a much higher cost. These findings lend credence to the notion that if people used antivirals effectively, making them available quickly on demand to private citizens could be a very effective way to control an outbreak.

## Introduction

We compare the performance of top-down versus bottom-up strategies in mitigating the spread of a simulated “Influenza-like illness” in Miami, Florida. A synthetic social network of Miami is used in which every person along with his/her social contacts is represented [1]. There are about 2.1 million people in Miami. In this model each person has an average of approximately 50 contacts, resulting in a social contact network of approximately 100 million edges. The median number of contacts is 42. An SEIR framework is used to represent the disease progression within the host. A brief description of the construction of the social contact network is given in the supporting information (Appendix S1). The disease model, the social network estimation and the interactive simulation engine (EpiFast) are described in detail in [2]–[5].

We consider how self-motivated individuals might react in the midst of an epidemic as they witness their immediate contacts become ill. We measure the impact of individual behavior and compare it with the impact of similar policies designed by public health officials and imposed top-down. There are several trade-offs between these strategies, e.g. individualistic behavioral modifications are often based on local information derived from one's immediate social network whereas public intervention is based on global information. Individuals react quickly and apply interventions immediately once their personal threshold is crossed whereas public health officials take longer to assess the situation and identify the appropriate intervention targets, often resulting in delay in implementing interventions.

The goal is to understand how individualistic actions, based on personal knowledge and beliefs, and aimed at self protection, fare in comparison to similar actions imposed by public policy makers who depend on private citizens' compliance.

This research compares the performance of three variants of ring strategies, a self-motivated, self imposed, “bottom-up” strategy and two “top-down” public health intervention strategies. A *ring strategy* typically consists of targeting all susceptible individuals in a local area around an outbreak of infectious disease. The area may be a concrete geographic area or, more abstractly, a set of neighbors in a contact network. The bottom-up strategy depends on each person's private awareness about the health state of his/her direct social contacts while the top-down strategy is based on public information about disease prevalence in a school or a census block group.

Previous studies have warned that ring strategies run the risk of “strategy failure” due to early depletion of an antiviral or vaccine stockpile. Given that the ring strategy considers both pre-exposure and post-exposure individuals for prophylaxis, it may not be efficient in using the limited stockpile of medical supplies. The standard ring strategy can result in premature and rapid usage of the existing stockpiles, leaving a population vulnerable to additional waves of the outbreak [6]. However, if the human-to-human transmission has a short incubation time, the ring strategy, GTAP (geographically targeted antiviral prophylaxis) strategy or another similar strategy may indeed be a very effective way to respond [7].

## Methods

### Disease Model

This study assumes that an “Influenza-like-illness” is being transmitted in the city of Miami, Florida through direct person-person contact. Note that Influenza may be transmitted via several pathways, such as contact with contaminated objects (fomites), as well as inhalation of aerosols containing virus particles. The relative contribution of each is largely an open question. Our model bases the probability of transmission on the duration of simultaneous presence in a small area.

The epidemic is seeded in five randomly chosen individuals. The model assumes that every day five new cases of this illness appear in addition to those generated by transmission. This kind of seeding of the epidemic helps control the level of variance across runs and ensures that each run will result in an epidemic. Realistically speaking, this assumption could represent infections imported from other regions of the country. The progression of disease in individuals is based on the usual SEIR model: at any given time, each individual in the population is in one of four health states : *susceptible*, *exposed*, *infectious*, or *removed* [1]–[3], [8]–[10].

- Everyone (except the seeds) starts in the susceptible state.
- After contact with an infectious person, a susceptible person enters the exposed state with probability 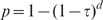, where 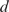 is the duration of contact and 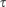 is the probability of transmission per unit of time.
- People remain in the exposed state for a certain number of days drawn from a discrete distribution of *incubation periods* with mean 1.9 days and standard deviation 0.49 day.
- People in the exposed state are not infectious.
- At the end of the incubation period, an exposed person becomes infectious and remains infectious a certain number of days drawn from a discrete distribution of *infectious periods* with mean 4.1 days and standard deviation 0.89 days. During this period, the exposed person may be *symptomatic* (with probability 1/3) or *asymptomatic* (with probability 2/3). An asymptomatic individual is 50% less likely to transmit the disease to others.
- A fraction of the symptomatic individuals, chosen uniformly at random, are observed to be infected. For the top-down strategies, this fraction corresponds to the fraction of symptomatic people who are diagnosed and thus known to the public health system; for the bottom-up strategy, this fraction corresponds to the fraction of one's symptomatic contacts whom one correctly identifies to be infected, regardless of whether they have been clinically diagnosed. For convenience, we refer to this fraction in either case as the diagnosis rate.
- Finally the individual becomes removed (or recovered) and remains so permanently.

### Mitigation Strategies

This research compares the performance of three strategies; one bottom-up and two top-down. The bottom-up strategy (*D1*) works almost like an inverse ring strategy. Under this strategy, self-motivated private citizens take action when the fraction of their direct contacts who are diagnosed exceeds a threshold. The top-down strategies are: (i) *Block*, take action on all people residing in a census block group if an outbreak is observed in the block group; and (ii) *School*, take action on all students in a school if an outbreak is observed in the school. The outbreak is assumed to be observed when the current fraction of people who are diagnosed exceeds a threshold. These are different from ring strategies like GTAP and others [7], [11] which consider a geographical ring around the detected person and treat the local population within the ring. In some cases of GTAP strategy, contact tracing methods were used to treat the identified index cases and provide prophylaxis (or vaccine) to the contacts of these index cases in predefined homogeneous mixing groups such as schools, households, workplaces, and neighborhood clusters. In all three strategies considered here, the experiments are conducted with outbreaks defined by threshold fractions of 1% and 5%. Note that in the bottom-up strategy, since the median number of contacts is 42, a threshold of 1% implies that more than half the compliant people will take action when they observe a single contact to be sick, more than 99% of the compliant people will take action when they observe 2 of their contacts to be sick; a threshold of 5% implies that almost half the compliant people will take action when they observe fewer than 3 contacts to be sick and almost 99% of the compliant will take action when they observe 8 of their contacts to be sick.

### Simulation Settings

Interventions to mitigate the epidemic include taking vaccines and antivirals, which decrease the probabilities of both infection and transmission if infected. The effectiveness of the interventions is compared using the following measures: (i) infection attack rate and (ii) the number of infections averted per drug course.

Vaccine and antivirals are chosen in our study because each can be applied at the public health level as well as an individual level. For example, during the 2009 H1N1 pandemic, people could go to clinics to get vaccinated or could obtain prophylaxis on demand, e.g. in Australia and New Zealand. Vaccines were also administered in an organized way, e.g. at schools.

Vaccines are often unavailable at the beginning of the epidemic, especially for new influenza strains. It takes time to identify the virus and prepare effective vaccines targeting the specific virus. Hence this study assumes that vaccines are available only from day 40 in the simulations. This is an optimistic assumption. Once available, the vaccines have a delay in becoming effective. We assume that they become effective after 2 weeks of application but remain effective for a long time – at least the duration of the outbreak. The total supply of vaccines and antivirals is assumed to be unlimited.

Antivirals become effective immediately but stay effective for only 10 days. Antivirals reduce the probability of infection upon exposure by 80% and the probability of transmission given infection by 87%. In the top-down case there is also a delay involved in applying the intervention to the entire *Block* or *School*. We consider two values for this delay: 1 day and 5 days. Other important parameters considered are the level of diagnosis and compliance. We assume diagnosis rates to be either 100% or 30% of symptomatic cases; and compliance rates to be either 100% or 50%. All results are reported based on the average of 25 replicates (simulation runs with a fixed set of parameter values). Table 1 gives an overview of all the parameters used in the experiment.

**Table 1:** Parameter choices.

1. **Strategies**: Direct contacts (D1), *Block*, *School*
2. **Interventions**: Antiviral (AV) and Vaccination (VAX)
3. **Transmissibility**: Low with 20% infection attack rate or high with 40% infection attack rate without intervention
4. **Diagnosis**: Probability of a symptomatic case being diagnosed and reported for public health interventions is 1.0 or 0.3 (i.e. 100% or 30%)
5. **Threshold value for taking actions**: 0.01 or 0.05. Under D1, the fraction of an individual's direct contacts observed to be infected; under Block (School), the fraction of people diagnosed in the block group (resp., school).
6. **Compliance rates**: 100% or 50%
7. **Delay in implementing interventions**: 1 day or 5 days delay for *Block and School*; B1, B5, S1 and S5 reflect 1 and 5 days delay for *Block and School* respectively. No delay in D1.
8. **Delay in effectiveness**: antivirals are immediately effective but vaccination becomes effective only after 2 weeks
9. **Duration of effectiveness**: Each course of antivirals is effective for 10 days and vaccination is effective until the end of the simulation.
10. **Simulation days and replicates**: 200 simulation days and 25 replicates.

The parameters, their interpretation, and values used in the experiments reported here. Parameters 1 - 7 are the factors included in a full factorial design experiment. The results are reported based on the average of 25 replicates for each cell in the design.

## Results and Discussion

The results are displayed in tables 2, 3, 4 and 5. The parameters *thres, diag, and comp* refer to threshold trigger value, probability of being diagnosed and the compliance rate respectively. AV and VAX represent antivirals and vaccines respectively. D1 is the bottom-up strategy, B1 is the top down *Block* strategy applied with 1 day implementation delay and B5 is applied with a 5 day delay. Similarly, S1 is the top down *School* strategy applied with 1 day implementation delay and S5 is applied with a 5 day delay.

**Table 2:** High transmissibility 7.35×10^−5^.

thres	diag	comp	attack rate (%, entry in bold if <10%)									
			AV					VAX				
			D1	B-1	B-5	S-1	S-5	D1	B-1	B-5	S-1	S-5
0.01	1.0	1.0	**0.3**	39.3	39.1	38.4	38.0	11.7	**3.8**	**5.2**	19.3	22.4
		0.5	**0.3**	39.4	39.1	38.8	38.5	12.7	**7.0**	**9.3**	25.2	27.5
	0.3	1.0	15.7	38.4	37.6	36.2	35.5	22.9	**9.9**	12.3	30.6	32.9
		0.5	17.2	38.5	38.0	37.4	37.0	23.8	13.7	17.0	33.4	35.0
0.05	1.0	1.0	24.4	37.8	37.0	36.0	35.8	32.7	15.0	18.1	36.3	37.4
		0.5	25.8	38.1	37.6	37.3	37.4	33.7	20.9	23.9	37.5	38.2
	0.3	1.0	35.9	35.7	34.2	39.9	39.9	38.7	26.3	29.2	40.0	40.0
		0.5	36.5	36.5	36.0	40.0	39.9	38.9	30.2	31.9	40.0	40.0

**Table 3:** Low transmissibility 5.35×10^−5^.

thres	diag	comp	attack rate (%, entry in bold if <5%)									
			AV					VAX				
			D1	B-1	B-5	S-1	S-5	D1	B-1	B-5	S-1	S-5
0.01	1.0	1.0	**0.2**	18.9	18.7	16.8	17.0	**2.9**	**2.6**	**2.9**	8.5	9.2
		0.5	**0.2**	19.4	19.3	18.0	18.0	**3.3**	**3.2**	**3.6**	10.0	10.7
	0.3	1.0	**0.8**	17.7	17.5	19.1	19.3	8.5	6.9	7.3	18.2	18.4
		0.5	**1.1**	18.5	18.5	19.5	19.6	8.9	7.8	8.5	18.6	18.8
0.05	1.0	1.0	7.0	17.6	17.2	20.0	20.1	16.0	10.2	11.0	20.1	20.1
		0.5	8.7	18.5	18.3	20.1	20.2	16.7	11.2	11.9	20.1	20.2
	0.3	1.0	17.9	19.9	19.9	20.2	20.3	19.6	19.2	19.3	20.2	20.3
		0.5	18.2	20.0	20.1	20.2	20.3	19.7	19.5	19.6	20.3	20.3

**Table 4:** High transmissibility 7.35×10^−5^.

thres	diag	comp	number of cases averted per drug course (entry in bold if >0.5)									
			AV					VAX				
			D1	B-1	B-5	S-1	S-5	D1	B-1	B-5	S-1	S-5
0.01	1.0	1.0	**10.19**	0.01	0.01	0.07	0.09	0.43	0.39	0.37	**0.89**	**0.75**
		0.5	**9.08**	0.01	0.02	0.10	0.13	0.40	**0.70**	**0.67**	**1.27**	**1.07**
	0.3	1.0	0.35	0.02	0.03	0.18	0.21	0.30	0.32	0.30	0.43	0.32
		0.5	0.32	0.03	0.04	0.25	0.28	0.29	**0.56**	0.50	**0.60**	0.45
0.05	1.0	1.0	0.37	0.03	0.04	0.28	0.27	0.15	0.34	0.30	0.22	0.15
		0.5	0.35	0.05	0.07	0.32	0.31	0.14	**0.54**	0.45	0.30	0.21
	0.3	1.0	0.29	0.06	0.08	0.31	0.15	0.09	0.20	0.16	0.12	0.12
		0.5	0.27	0.10	0.12	0.25	0.35	0.08	0.30	0.25	0.25	0.11

**Table 5:** Low transmissibility 5.35×10^−5^.

thres	diag	comp	number of cases averted per drug course (entry in bold if >0.5)									
			AV					VAX				
			D1	B-1	B-5	S-1	S-5	D1	B-1	B-5	S-1	S-5
0.01	1.0	1.0	**8.33**	0.01	0.01	0.16	0.15	**0.68**	0.19	0.18	**0.60**	**0.54**
		0.5	**7.96**	0.08	0.01	0.19	0.19	**0.60**	0.35	0.34	1.00	**0.91**
	0.3	1.0	**5.10**	0.03	0.03	0.17	0.14	0.42	0.17	0.16	0.34	0.31
		0.5	**4.06**	0.04	0.04	0.20	0.15	0.40	0.30	0.28	**0.51**	0.43
0.05	1.0	1.0	**1.29**	0.04	0.05	0.00	0.00	0.21	0.15	0.14	0.00	0.00
		0.5	**1.02**	0.05	0.05	0.00	0.00	0.18	0.27	0.24	0.00	0.00
	0.3	1.0	0.39	0.03	0.03	0.00	0.00	0.08	0.16	0.14	0.00	0.00
		0.5	0.35	0.00	0.00	0.00	0.00	0.06	0.18	0.16	0.00	0.00

Tables 2 and 3 show that antivirals are very effective under the bottom-up (D1) strategy but have marginal effect under the two top-down strategies. Under the bottom-up strategy, antivirals can drop the attack rate from the original 20%, in the case of low transmissibility, and 40% in the case of high transmissibility, to less than 1% in both cases. In the top-down strategies the attack rate stays close to the 20% and 40% levels under both low and high transmissibility respectively. If the threshold trigger changes from 1% diagnosed to 5% diagnosed, i.e. if the anti-viral intervention is implemented late, then the bottom-up strategy results in high attack rate under both high and low transmissibility. However, it still performs better than either top-down strategy. These results show that the performance of the bottom-up AV strategy is robust to delay in implementation, drop in compliance rate and increase in the threshold value under the parameter settings considered here.

The *Block* and *School* strategies also result in high cost and high depletion of antivirals because significantly more people have to be given antivirals than in case of D1. Tables 4 and 5 display the number of infections averted per drug course. The *Block* and *School* strategies avert less than one case per drug course whereas the D1 strategy averts up to 10 cases per drug course. These experiments support the claim that if people can be trusted with the proper use of antivirals, their “over-the-counter” or “on-demand” availability to private citizens can be an effective way to control the disease.

Vaccination performs better under the *Block* strategy than under the *School* strategy, regardless of the level of transmissibility. Given a two week delay in vaccines becoming effective, cases in one's immediate neighborhood become less relevant. The *Block* strategy is able to form a larger ring around “hot spots”. However, significantly more vaccines are needed to support the *Block* strategy. The D1 strategy performs better than the *School* but worse than *Block* in terms of attack rate; however, it uses the least number of vaccines. It also averts more cases per drug course in case of low transmissibility. The *School* strategy is able to avert more cases per vaccine than the *Block* strategy but results in higher attack rate. The experimental results imply that, under the scenarios considered here, the *Block* strategy would work best if a sufficient number of vaccines were available.

Tables 2 and 3 show that the attack rates are not very different for B1 vs. B5 and S1 vs. S5, i.e. 1 day or 5 day delay does not really affect the performance of the top-down strategies. Given the overall low effectiveness of the top-down strategies, it is not surprising to see that the differences in compliance rate, diagnosis rate, and threshold trigger have no significant effect on the number of cases averted per drug course, see tables 4 and 5.

We also note that drug consumption under AV and VAX changes linearly with compliance. A higher compliance rate results in lower attack rate, but the attack rate does not decrease linearly with increased consumption. The attack rate decreases at a lower rate than the increase in consumption rate. Note that if the diagnosis rate is low and the threshold for intervention is high, no intervention is effective in controlling the attack rate, see the last row of tables 2, 3, 4 and 5.

**Figure 1:**
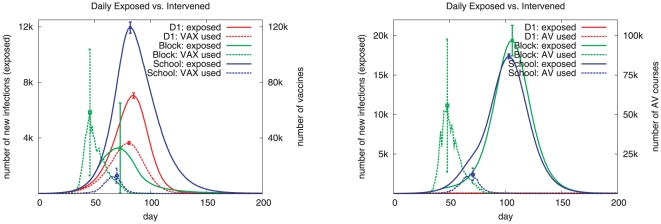
Number of people exposed versus the amount of vaccine and antiviral used under each of the three strategies considered. On the left, number exposed versus the number of vaccines used on a daily basis; on the right, the number exposed versus the courses of antivirals used on a daily basis. Error bars at the peak of each curve show the standard deviation over 25 runs of the stochastic simulation and are indicative of the level of error over the rest of the curve. *D1* refers to the bottom-up strategy, and *Block and School* refer to the top-down strategies. The parameters settings used here include high transmissibility i.e. 40% infection attack rate, diagnosis probability of 1, threshold value of 0.01, and the compliance probability of 0.5.

**Figure 2:**
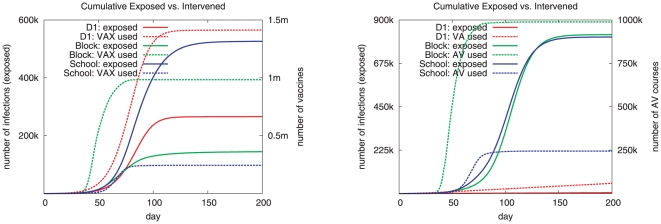
Cumulative number of people exposed versus the cumulative amount of vaccine and antiviral used under each of the three strategies considered. On the left, cumulative number exposed versus the cumulative number of vaccines used; on the right, the cumulative number exposed versus the cumulative courses of antivirals used. *D1* refers to the bottom-up strategy, and *Block and School* refer to the top-down strategies. The parameters settings used here include high transmissibility i.e. 40% infection attack rate, diagnosis probability of 1, threshold value of 0.01, and the compliance probability of 0.5.

Figure 1 illustrates the trade-off across the three strategies for VAX and AV respectively. Each panel shows the epidemic curves marked by solid lines and the number of drug courses used by dotted lines for each strategy. Ideally one would like to see a strategy where both the attack rate and the number of courses used are small. In the VAX case, none of the strategies show this. Under the *School* strategy, the number of doses of vaccine used is the smallest but the attack rate is the highest. Under the *Block* strategy, the attack rate is the smallest but the number of doses of vaccine used is very high. The *D1* strategy has an intermediate attack rate and intermediate number of doses of vaccine used. Figure 1 shows that D1 is a clear winner as it has the lowest attack rate and the lowest number of anti-viral courses used. Similar conclusions are reflected in the cumulative plots, Figure 2, which shows the cumulative number of people exposed versus the cumulative amount of vaccines and antivirals used under each of the three strategies.

Depending upon public health policy goals and the availability of antivirals and vaccines, each of these strategies can be important. The experimental scenarios considered here suggest the following: if the transmissibility is high and vaccines are available in abundant supply, the *Block* strategy is likely to be the best choice. On the other hand, if only antivirals are available and only in limited amount, one might consider distributing them to private citizens on-demand or over-the-counter to make them quickly and easily available. If antivirals and vaccines are both available only in limited quantities, identification of infectious cases is administratively expensive, and compliance with a public policy is an issue, it would be best to motivate individuals to self-intervene by applying D1.

This study examines the comparative effectiveness of self-driven behavioral interventions and publicly imposed interventions using a parametrized experimental design. For a realistic set of parameters, it demonstrates how individual behavioral modifications can result in controlling the attack rate in a much more cost effective way than either of the public policies that are imposed top down. The novelty of this research lies in the fact that the private citizens use local information derived from the health status of their immediate social contact network to determine the time of intervention. The outcomes of this behavior and its comparison with the top-down policies have never been studied before.

## Supporting Information

Appendix S1 Construction of social contact networks.
(PDF)

## References

[1] Bisset K, Chen J, Feng X, Kumar VSA, Marathe M (2009). EpiFast: A fast algorithm for large scale realistic epidemic simulations on distributed memory systems.. Proceedings of the 23^rd^ International Conference on Supercomputing (ICS).

[2] Barrett C, Bisset K, Eubank S, Feng X, Marathe M (2008). Episimdemics: an efficient algorithm for simulating the spread of infectious disease over large realistic social networks..

[3] Barrett C, Bissett K, Leidig J, Marathe A, Marathe M (2010). An integrated modeling environment to study the co-evolution of networks, individual behavior and epidemics.. AI-Magazine.

[4] Bisset K, Marathe M (2009). A cyber-environment to support pandemic planning and response.. DOE SciDAC Magazine.

[5] Eubank S, Guclu H, Kumar VA, Marathe M, Srinivasan A (2004). Modeling disease outbreaks in realistic urban social networks.. Nature.

[6] Balicer RD, Huerta M, Davidovitch N, Grotto I (2005). Cost-benefit of stockpiling drugs for influenza pandemic.. Emerging Infectious Diseases.

[7] Longini I, Nizam A, Xu S, Ungchusak K, Hanshaworakul W (2005). Containing pandemic influenza at the source.. Science.

[8] Bailey N (1975). The Mathematical Theory of Infectious Diseases and Its Applications..

[9] Kuznetsov Y, Piccardi C (1994). Bifurcation analysis of periodic SEIR and SIR epidemic models.. Journal of Mathematical Biology.

[10] Murray JD (2002). Mathematical Biology I. An Introduction, volume 19 of Biomathematics..

[11] Ferguson N, Cummings D, Fraser C, Riley S, Meeyai A (2005). Strategies for containing an emerging influenza pandemic in southeast asia.. Nature.

[12] Beckman RJ, Baggerly KA, McKay MD (1996). Creating synthetic base-line populations.. Transportation Research A – Policy and Practice.

[13] Barrett C, Beckman R, Berkbigler K, Bisset K, Bush B (2001). TRANSIMS: Transportation analysis simulation system..

