# Supplementary material for: Comparing Effectiveness of Top-Down and Bottom-Up Strategies in Containing Influenza
Source: PLoS One. 2011 Sep 22;6(9):e25149. doi: 10.1371/journal.pone.0025149 (PMC3178616; doi:10.1371/journal.pone.0025149)
Supplement: Appendix S1 — Construction of social contact networks. (PDF) [file pone.0025149.s001.pdf]

Appendix S1: Construction of social contact networks

We have created synthetic social contact networks for the entire United States, where more than 300 million network nodes represent individuals and the edges represent daily contacts between the individuals [1–3]. These networks are used in agent based simulations to study phenomena such as the propagation of contagious diseases, spread of rumors, peer influences etc. The approach to constructing social contact networks consists of four steps [4]:

1. **population synthesis**, in which a synthetic representation of each person and household in a region is created from the US census data;

2. **activity assignment**, in which each synthetic person in a household is assigned a set of activities to perform during the day, along with the times when the activities begin and end, as given by activity or time-use survey data;

3. **location choice**, in which an appropriate real location is chosen for each activity for every synthetic person based on data such as tax data, land-use data or Dunn and Bradstreet data;

4. **contact estimation**, in which each synthetic person is deemed to have made contact with a subset of other synthetic people simultaneously present at the same location.

The resulting social contact network is a graph whose vertices are synthetic people, labeled by their demographics, and whose edges represent contacts determined in step four, labeled by the duration of contact and type of activity. Note that it is *impossible* to build such a network by simply collecting field data; the use of data-driven, but generative, models to build such networks is a unique feature of this work.

The population synthesis process preserves the confidentiality of the individuals in the original data sets, yet produces realistic attributes and demographics for the synthetic individuals. Joint demographic distributions are reconstructed from the marginal distributions available in typical census data together with joint distributions in Public Use Microdata Samples (PUMS) using an iterative proportional fitting technique. This technique guarantees that a census of our synthetic population is statistically indistinguishable from the input census when aggregated to block groups.

Particularly important is step 2, in which each synthetic household is assigned a set of activities to perform during the day. A set of activity templates for households is synthesized, based on several thousand responses to an activity or time-use survey. The activities pool all survey data and thus represent a single normative day. Each synthetic household is then matched with one of the survey households, using a decision tree based on demographics such as the number of people in the household, number of children of various ages, income, etc. The synthetic household is assigned the activity template of its matching survey household. This modeling methodology, *activity based travel demand modeling* is the *de facto* standard in transportation science [4, 5].

In step 3, each activity performed by each member of each household is assigned a location using a gravity model based on observed land-use patterns, tax data, etc.

In step 4, a social network is generated based on co-location of people at various times throughout the day. The *dynamic social contact network* is represented by a vertex- and edge-labeled graph. If two people are simultaneously present in a location, there is an edge between them in the graph, labeled by the duration of their co-location.

References

1. Barrett C, Bissett K, Leidig J, Marathe A, Marathe M (2010) An integrated modeling environment to study the co-evolution of networks, individual behavior and epidemics. AI-Magazine : 75-87.

2. Bisset K, Marathe M (2009) A cyber-environment to support pandemic planning and response. DOE SciDAC Magazine : 36-47.

3. Eubank S, Guclu H, Kumar VA, Marathe M, Srinivasan A, et al. (2004) Modeling disease outbreaks in realistic urban social networks. Nature 429: 180–184.

4. Beckman RJ, Baggerly KA, McKay MD (1996) Creating synthetic base-line populations. Transportation Research A – Policy and Practice 30: 415-429.

5. Barrett C, Beckman R, Berkbigler K, Bisset K, Bush B, et al. (2001) TRANSIMS: Transportation analysis simulation system. Technical Report LA-UR-00-1725. An earlier version appears as a 7 part technical report series LA-UR-99-1658 and LA-UR-99-2574 to LA-UR-99-2580, Los Alamos National Laboratory Unclassified Report.
